# Immunogenicity and waning immunity from the oral cholera vaccine (Shanchol^™^) in adults residing in Lukanga Swamps of Zambia

**DOI:** 10.1371/journal.pone.0262239

**Published:** 2022-01-05

**Authors:** Harriet Ng′ombe, Michelo Simuyandi, John Mwaba, Charlie Chaluma Luchen, Peter Alabi, Obvious Nchimunya Chilyabanyama, Cynthia Mubanga, Luiza Miyanda Hatyoka, Mutinta Muchimba, Samuel Bosomprah, Roma Chilengi, Geoffrey Kwenda, Caroline Cleopatra Chisenga

**Affiliations:** 1 Center for Infectious Disease Research in Zambia, Lusaka, Zambia; 2 Department of Biomedical Sciences, School of Health Sciences, University of Zambia, Lusaka, Zambia; 3 Department of Biostatistics, School of Public Health, University of Ghana, Accra, Ghana; Public Health England, UNITED KINGDOM

## Abstract

**Introduction:**

In cholera endemic areas, the periodicity of cholera outbreaks remains unpredictable, making it difficult to organize preventive efforts. Lack of data on duration of protection conferred by oral cholera vaccines further makes it difficult to determine when to deploy preemptive vaccination. We report on the immunogenicity and waning of immunity to Shanchol^™^ in Lukanga Swamps.

**Methods:**

We enrolled a cohort of 223 participants aged between 18 and 65 years old from whom serum samples were collected at baseline, day 28 before administration of the second dose, and consecutively at 6, 12, 24, 30, 36, and 48 months. Vibriocidal antibody titres were measured and expressed as geometric mean titres. Box plots and 95% CI were computed at each visit for both Inaba and Ogawa. Seroconversion was defined as a four fold or greater increase in antibody titres compared to baseline titres.

**Results:**

Overall, seroconversion against *V*. *cholerae* Inaba and Ogawa after 1st dose was 35/134 (26%) and 34/134 (25%) respectively. We observed a statistical difference in seroconversion between the two subgroups of baseline titres (low <80 and high ≥80) for both Inaba (p = 0.02) and Ogawa (p<0.0001). From a baseline of 13.58, anti-Ogawa GMT increased to 21.95 after the first dose, but rapidly waned to 14.52, 13.13, and 12.78 at months 6, 12 and 24 respectively, and then increased to 13.21, 18.67 and 23.65 at months 30, 36 and 48 respectively. A similar trend was observed for anti-Inaba GMT across the same time points.

**Conclusion:**

We found that Shanchol^™^ was immunogenic in our study population and that vibriocidal antibodies may not be a good marker for long-term immunity. The observed rise in titres after 36 months suggests natural exposure, and this may be a critical time window opening for natural transmission in an endemic areas. We recommend re-vaccination at this time point in high risk areas.

## Introduction

Cholera is a debilitating diarrhoeal disease caused by an invasion of the body with the bacterium *Vibrio cholerae* of serogroups O1 and O139, with the former having serotypes Inaba, Ogawa and Hikojima [1, 2]. It is a serious global health threat in developing countries where cholera infects about 3.5 million yearly [3] and a growing concern in endemic countries. It accounts for approximately 2.86 million cases and 95,000 deaths annually [4].

The re-emergence of cholera in Africa during the seventh pandemic resulted in 40 million cholera cases in African countries between 1970 and 2017 [5–7]. According to the World Health Organisation (WHO), African countries reported 179,835 and 3,220 cholera cases and deaths, respectively [5]. The case fatality ranged from 0 in many countries to 3.2% in Zambia, 5.2% in Angola and 6.8% in Chad [5]. The most recent cholera outbreaks in Zambia (2017–2018) resulted in 5900 cases and 114 deaths [8]. In 2016, Lukanga Swamps reported 27 cases with 7.4% fatality rate [9]. The high case fatality rate mirror major constraints in access to case management in Africa.

The evolving epidemiology of disease and an increase of at-risk areas have led to embracing the use of a multisectoral approach [10] to prevent cholera recurrence in hotspot areas. This approach includes, among others, the use of Oral Cholera Vaccine (OCV). There are currently three Oral Cholera Vaccines pre-qualified by WHO and includes Dukoral, an inactivated whole-cell recombinant B subunit and both Euvichol and Shanchol^™^ which are also inactivated whole-cell vaccines consisting of *V*. *cholerae* O1 (El Tor and classical biotypes) and O139 serogroups. Despite the use of two-dose regimen, which is well known for preventing cholera, cholera still reoccurs in areas where vaccines have been deployed [11]. We postulate that this may be attributable to the waning of immunity conferred by the vaccine as well as poor vaccine coverage. Therefore, the need for continued use of OCVs and administration of booster doses in high-risk areas cannot be over emphasised for effective prevention and control of cholera.

The limited data on the duration of protection by oral cholera vaccines renders it difficult to determine the ideal timing for the deployment of booster doses of the vaccine. Therefore, longitudinal studies of vaccine immunogenicity are important to determine the waning of immunity, risk of vaccination failure in individuals and to verify whether recommended dosing regimens guarantee long-lasting immunity.

We evaluated the immunogenicity and waning of vibriocidal antibodies following administration of a 2-dose regimen of Shanchol^™^ in individuals residing in a cholera endemic area of Lukanga Swamps, Zambia.

## Materials and methods

### Study area

The study was conducted in Lukanga which has a population of approximately 16,000 people and is located in Kapiri-Mposhi district of Zambia. Lukanga is a swampy area surrounded by large water bodies and divided into several lagoons. The Swamps that serve as drinking water sources are often marred with waste disposal including defecation. The main livelihood for individuals residing in the Swamps is fishing. Annual outbreaks of cholera were reported in Lukanga Swamps in years prior to deployment of the vaccine (Shanchol^™^) in 2016, and as such the area is listed among cholera hotspots in Zambia.

### Study design and enrolment of study participants

**Fig 1:**
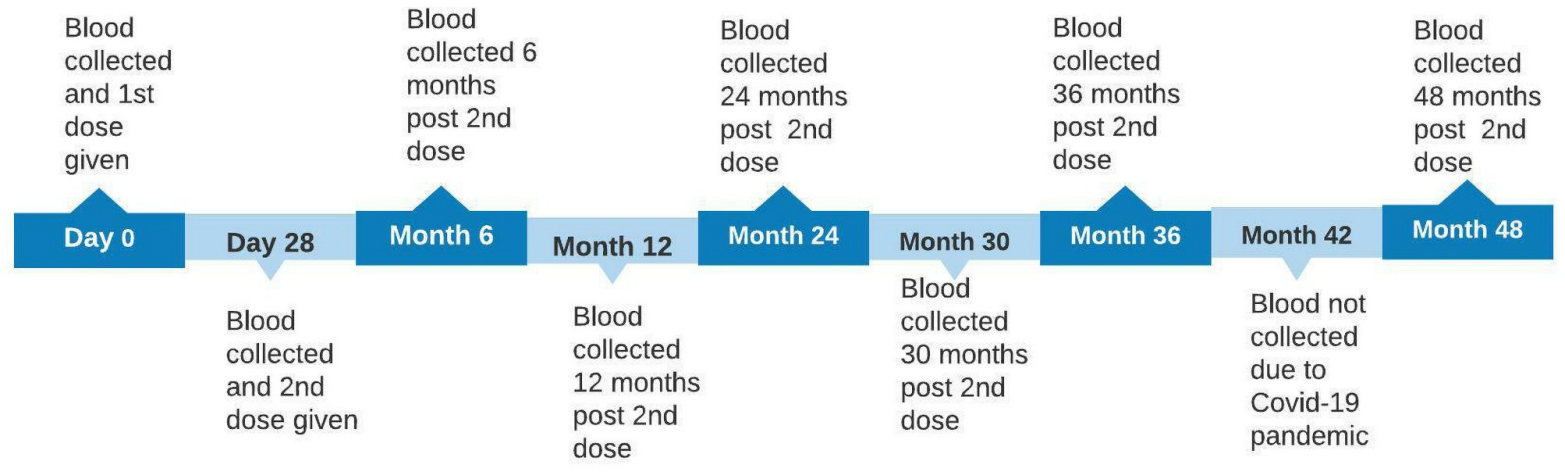
Day 0 (baseline), 1st dose of Shanchol was administered followed by 2nd dose at day 28 while months 6, 12, 24, 30, 36 and 48 where follow up visits.

We conducted a longitudinal study and enrolled 223 adults aged between 18 to 65 years old that consented to be followed up for a period of 48 months. Clinical and demographic data was collected from all consenting adults. The exclusion criteria included pregnancy, history of hospitalisation due to cholera or any acute illness. Approximately 10 mls of blood was collected at baseline (day 0) before receipt of first dose, at day 28 before receiving the second dose, and at 6, 12, 24, 30, 36 and 48 months (Fig 1). All samples were centrifuged at 3000 rpm for 20 minutes and collected serum was aliquoted and stored at -80°C pending testing.

### Sample size considerations

The minimum required sample size was 176, calculated with a 95% CI (α = 0.05 (two tailed)), power 80% (β = 0.2), estimated difference of 0.3 log titers and conservative estimates of 0.5 variance for pre-vaccine and post-vaccine groups. Due to marginal transience note in the population (Lukanga swamps), we inflated the sample size by 20% to account for attrition yielding a required sample size of 212 participants.

### Laboratory methods

Two doses of OCV Shanchol^™^ manufactured by Shantha Biotechnics Private Limited, India (Shantha Biotechnics Limited) was administered to the participants 28 days apart; blood samples were collected at the consecutive visit time points. The vibriocidal assay was used to screen for cholera antibody titres pre and post-vaccination. This assay was performed as described previously [12] with a few modifications. *V*. *cholerae* O1 Inaba (EDVRU/ZM/091-10) and Ogawa (EDVRU/ZM/2016) were used. These strains were quality checked at Johns Hopkins University in the United States and their performance was comparable to standard strains of Inaba (T19479) and Ogawa (X25049). Briefly, colonies from overnight cultures were inoculated in Brain Heart Infusion (BHI) Broth and incubated at 37°C for about 4 hours before harvesting the cells. Heat inactivated serum, exogenous guinea pig complement (Sigma Aldrich S1639-5ML) and *V*. *cholerae* bacterial cells were then put in 96 well microtitre tissue culture plates (Life sciences, Durham, USA) and incubated at 37°C. Vibriocidal titres were defined as the 595 nm reciprocal of the highest serum dilution resulting in a 50% reduction in optical density read at compared to positive control wells without serum. Seroconversion was defined as a 4-fold or greater increase from the baseline vibriocidal titres [13]. A standard monoclonal antibody (mAb) and a high titres standard serum [14] were used to normalise the results in case of inter-assay variations.

### Statistical analysis

Participants’ socio-demographic and clinical characteristics were presented as frequencies (percentages) and mean (standard deviation) for categorical and continuous variables respectively. Vibriocidal antibody titres were expressed as geometric mean titres (GMT), and where computed for each key background characteristics. We determined the GMTs which were plotted as box plots and 95% CI at each visit for both Inaba and Ogawa. The seroconversion rate was defined as the percentage of participants with at least a four-fold rise in serum vibriocidal antibody titres from baseline to 28 days post vaccination. We compared association between seroconversion, for Inaba and Ogawa, and background characteristics using Fisher’s exact test and crude odds ratio. We also compared seroconversion rates between baseline titre groups (low <80 and high ≥80) as previously described [15, 16]; this was to ascertain the influence of pre-existing antibodies on antibody immune response after vaccination. The level of statistical significance was set at a 2-tailed p-value of 0.05 or less. Data analysis was performed using Stata 16.0 for Windows (Stata Corp, College Station, TX, USA).

### Regulatory and ethics statement

All study staff were trained on study methodology and human participants’ research ethics, and written informed consent was obtained from all participants prior to any study procedures. The study protocol was reviewed and approved by the University of Zambia Biomedical Ethics Committee (UNZABREC #: 007-12-16) and the National Health Research Authority (NHRA#: MH/101/23/10/1). The vaccine was provided by the Ministry of Health in Zambia, and this study was registered on Clinical Trials.gov with trial # NCT04423159.

## Results

**Fig 2:**
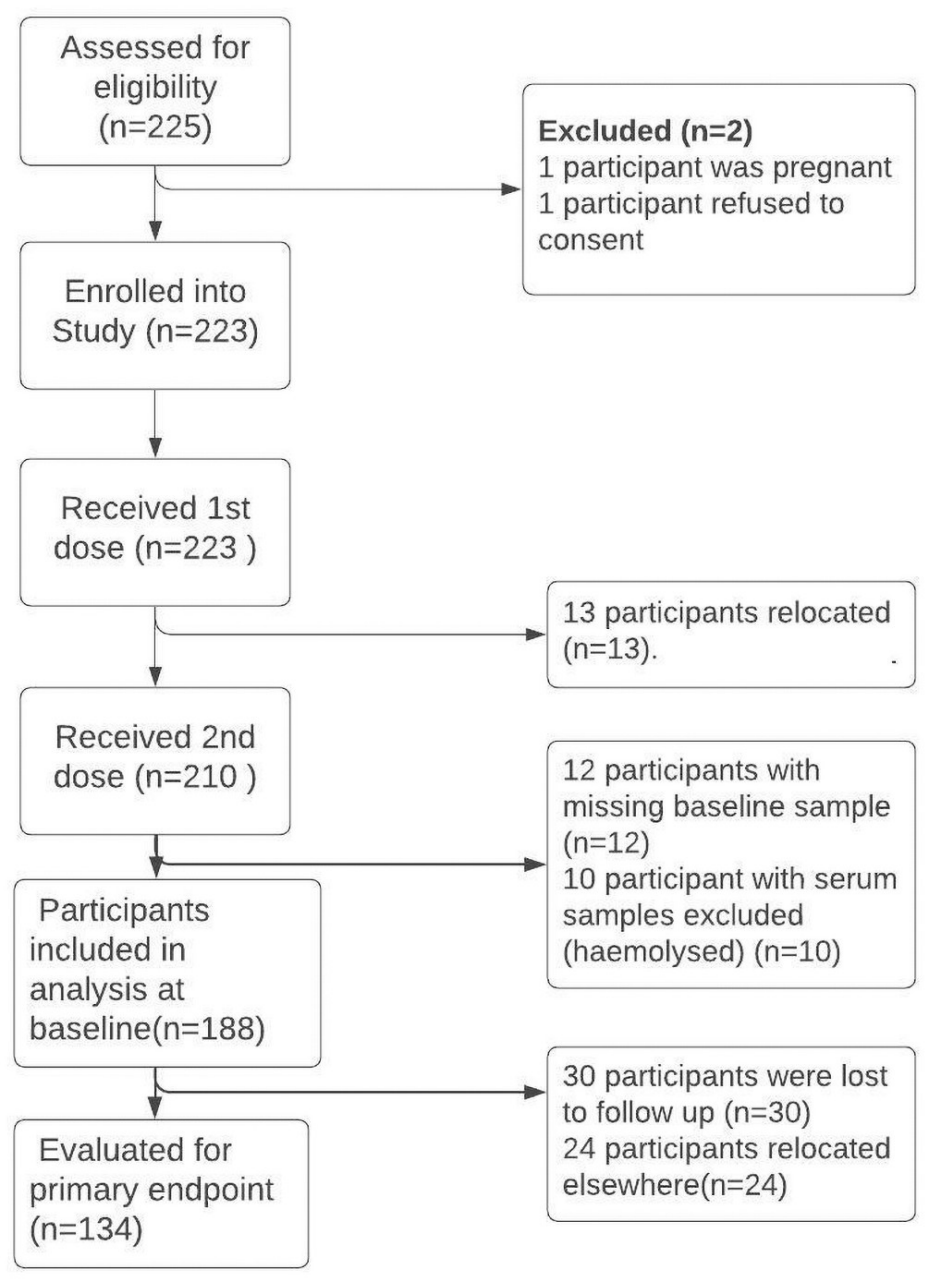
Participant flow diagram.

Enrollment took place between October and November 2016. A total of 225 individuals were screened and 223 satisfied the inclusion criteria and were enrolled (Fig 2). The majority of the participants (92%) were males (Table 1). The median age was about 38 years (IQR = 26–45), and most (71%) of participants completed their education only up to grade seven (primary education). Most of the participants were engaged in fishing (76%) and up to 76% also had the Swamps as the main source of drinking water and toilet facility.

**Table 1:** Geometric Mean Titres (GMTs) of *V*. *cholerae* O1 Ogawa and Inaba by background characteristics.

Characteristics	Number (% of total)	Ogawa	Inaba
		GMTs (95% CI)	GMTs (95% CI)
**Age (years)** *			
16–24	38 (20.3)	17.52 (13.83–22.19)	12.62 (10.31–15.44)
25–34	39 (20.9)	13.61 (11.27–16.44)	11.01 (9.07–13.36)
>34	110 (58.8)	16.10 (14.24–18.20)	10.87 (9.83–12.01)
Median (IQR)	38 (26–45)		
**Sex**			
Female	16 (8.5)	17.76 (12.90–24.45)	10.07 (8.11–12.51)
Male	172 (91.5)	15.51 (14.06–17.12)	11.39 (10.44–12.43)
**Education** *			
Grade 1–7	104 (70.8)	18.75 (16.35–21.27)	11.91 (10.68–13.27)
Grade 8–12	43 (29.2)	12.76 (10.60–15.35)	11.63 (9.57–14.14)
**Occupation** *			
Fishing	130 (75.6)	16.10 (14.38–18.03)	11.99 (10.81–13.30)
Trading	32 (18.6)	18.29 (14.13–23.68)	10.10 (8.57–11.91)
Others	10 (5.8)	11.49 (8.54–15.45)	8.83 (6.37–12.33)
**Source of drinking water** *			
Borehole/well	40 (24.2)	17.31 (13.80–21.71)	10.42 (8.91–12.18)
Swamps	125 (75.8)	16.28 (14.51–18.27)	11.60 (10.44–12.89)
**Toilet type** *			
Swamp/bush	124 (76.5)	15.94 (14.19–17.90)	11.79 (10.59–13.11)
Pit latrine/ toilet	38 (23.5)	17.69 (14.09–22.21)	9.66 (8.40–11.11)
**Total**	188 (100.0)	15.72 (14.31–17.27)	11.25 (10.37–12.21)

* Total not equal to 188 due to missing information, GMT: Geometric Mean Titres, CI: Confidence Interval

Seroconversion rate against *V*. *cholerae* O1 Ogawa and O1 Inaba was 25% and 26% respectively as shown in Table 2. Those who were >34 years old had a comparable seroconversion rate (56%, p = 0.309 and 57.1%, p = 0.355) with the younger participants against the Ogawa and Inaba serotype respectively. Fishermen where observed to have a higher seroconversion rate compared to participants engaged in other occupations such as trading, though this association was not significant.. Interestingly, we found a significant difference in seroconversion rates between the two subgroups of baseline titres (low <80 and high ≥80) for both serotypes Ogawa (<0.001) and Inaba (0.021).

**Table 2:** Seroconversion against *V*. *cholerae* O1 Ogawa and Inaba (n = 134).

	Ogawa			Inaba		
Characteristics	Total n (%)	n (%) Seroconverted	p value	Total n (%)	n (%) Seroconverted	p value
**Age (years)**						
16–24	27(20.1)	8(29.6)	0.309	27(20.3)	8(29.6)	0.355
25–34	30(22.4)	8(26.7)		29(21.8)	8(27.6)	
>34	76(56.7)	17(22.4)		76(57.1)	17(23.7)	
Missing*	1(0.7)	1(100)		1(0.8)	1(100)	
**Sex**						
Female	13(9.7)	2(15.4)	0.384	13(9.8)	1(7.7)	0.108
Male	121(90.3)	32(26.4)		120(90.2)	34(28.3)	
**Education**						
Grade 1–7	72(53.7)	18(25)	0.827	72(54.1)	21(29.2)	0.72
Grade 8–12	35(26.1)	8(22.9)		35(26.3)	8(22.9)	
Missing*	27(20.1)	6(29.6)		26(19.5)	8(23.1)	
**Occupation**						
Fishing	90(67.7)	22(24.4)	0.229	90(67.7)	21(23.3)	0.635
Trading	24(17.9)	4(16.7)		24(18)	7(29.2)	
Others	10(7.5)	3(30)		10(7.5)	4(40)	
Missing*	10(7.5)	5(50)		9(6.8)	3(33.3)	
**Baseline Titre**						
Low (<80)	110(82.1)	34(30.9)	<0.001	121(90.3)	35(28.9)	0.021
High (≥80)	24(17.9)	0(0)		12(9)	0(0)	
**Total**	**134(100)**	**34(25.4)**		**133(100)**	**35(26.3)**	

Fisher exact test was used to compare seroconversion by different baseline characteristics,

*Demographic information was not available.

Participants older than 34 years had higher odds of seroconverting to O1 Ogawa respectively though this was not significant (OR = 1.26 (0.5–3.34); p-value = 0.6391 & OR = 1.46 (0.5–3.92); p-value = 0.6391). Males were 49% less likely to seroconvert to Ogawa (OR = 0.51 (0.1–2.41); p-value = 0.392). Having different levels of education did not seem to influence the odds of seroconverting to the vaccine. Similar to Ogawa, socio-demographics did not affect seroconversion against Inaba significantly (Table 3).

**Table 3:** Odds of seroconversion to *V*. *cholerae* Ogawa and Inaba (n = 134).

	Ogawa		Inaba	
	Crude OR (95% CI)	p value	Crude OR (95% CI)	p value
**Gender**				
Male	ref	0.392	ref	0.142
Female	0.51 (0.1–2.41)		0.21 (0–1.68)	
**Age**				
35 and above	ref	0.6391	ref	0.679
25–34 years	1.26 (0.5–3.34)		1.23 (0.5–3.24)	
16–24 years	1.46 (0.5–3.92)		1.36 (0.5–3.62)	
**Education**				
grade 1–7	ref	0.808	ref	0.492
grade 8–12	0.89 (0.3–2.3)		0.72 (0.3–1.84)	
**Occupation**				
Fishing	ref	0.42	ref	0.486
Traders	0.62 (0.2–2)		1.35 (0.5–3.7)	
Others	1.32 (0.3–5.57)		2.19 (0.6–8.5)	

**Fig 3:**
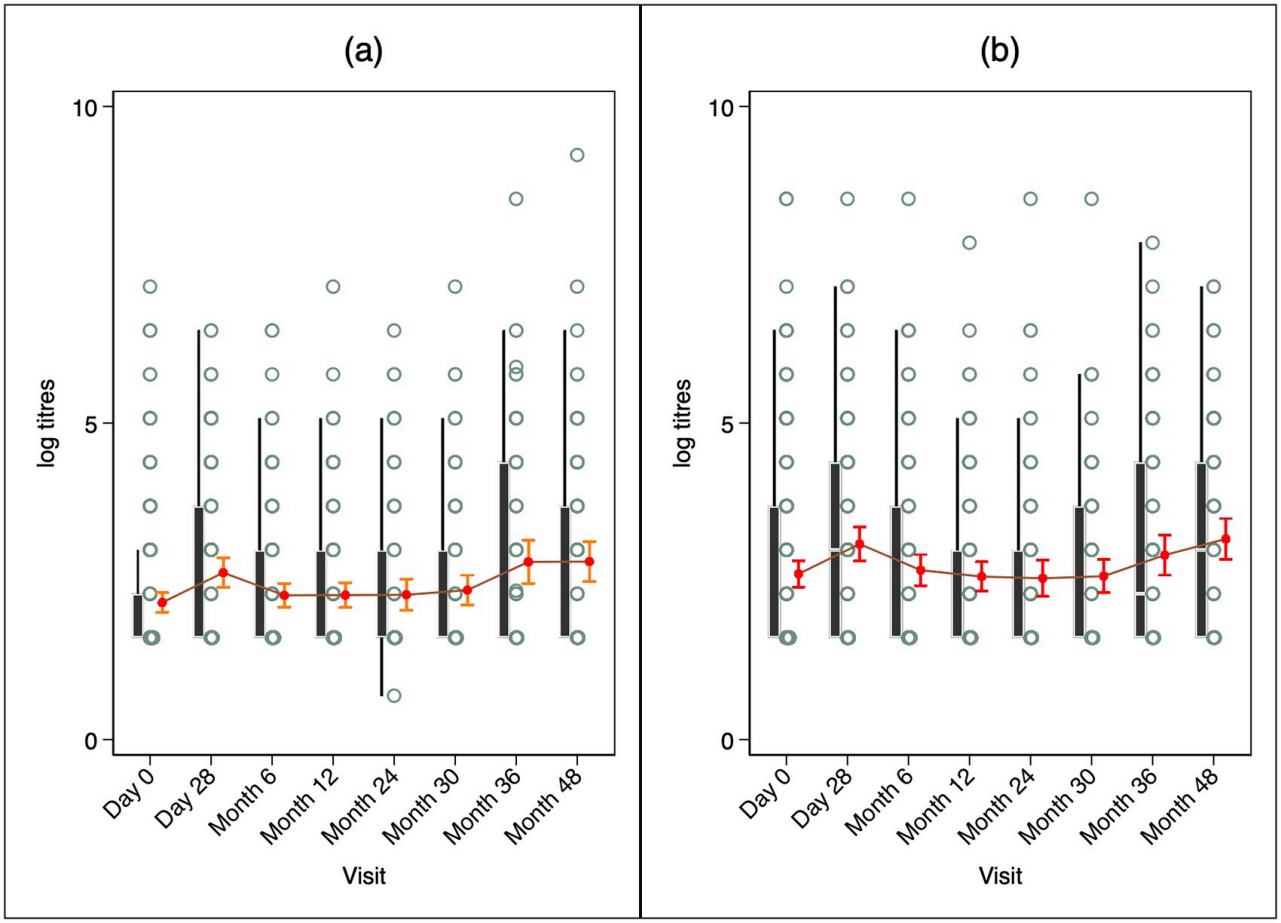
Kinetics of vibriocidal GMTs expressed as box plots and 95% confidence intervals (a) V. cholerae O1 Inaba (b) V. cholerae Ogawa. There was a rise in antibody titres in both serotypes at day 28 and then started to decline close to baseline titres at month 12. A significant rise was observed at months 36 and 48.

Baseline GMTs for Ogawa was 13.58(10.88–16.95) and increased markedly to 21.95(16.76–28.76) on Day 28. However, GMTs dropped to 14.52(11.37–18.56), 13.13(10.41–16.56), and 12.78(9.59–17.17.04) at 6, 12 and 24 months respectively before it was marginally increased to 13.21(10.17–17.17), 18.67(13.56–25.72) and 23.65(17.09–32.73) at months 30, 36 and 48 as shown in Fig 3. From a baseline of 8.95(7.56–10.60), anti-Inaba GMT increased to 13.96(11.06–17.62) after the first dose, but rapidly began to wane to 9.76(8.10–11.77), 9.79(8.04–11.91), and 9.84(7.69–12.59) at months 6, 12 and 24 respectively, and then increased to 10.60(8.39–13.40), 16.55(11.17–23.28) and 16.82(12.22–23.15) at months 30, 36 and 48 respectively.

## Discussion

Seroconversion is considered a proxy for protection. In our study, we found that Shanchol was immunogenic with 35(26%) and 34(25%) of participants seroconverting to Inaba and Ogawa respectively. While the vaccine was found to be immunogenic, our results show lower seroconversion rate than that reported in other regions [17–20]; though these studies assessed for seroconversion at day 14 post vaccination. The possible explanation for low seroconversion rate in our cohort could be because of preexisting immunity as evidenced by the high baseline titres in both Inaba (9%) and Ogawa (18%). South Sudan recorded 72% seroconversion rate at 14^th^ day post 1^st^ dose with majority of seroconverts having low baseline vibriocidal titres (<80) [21]. Consequently, Saha et al also reported that in Bangladesh 86% of participants with high baseline titres did not seroconvert to the vaccine [18]. High vibriocidal antibody titres prior vaccination have been linked with lower seroconversion rate, it has been observed that following primary exposure, the microfold (M cells) prevent vibrio antigen from penetrating the epithelial wall resulting in lack of secondary response. We postulate that other factors such as nutrition, environmental enteric dysfunction (EED) and co-infections might have contributed to the lower immunogenicity like what has been observed in live oral cholera vaccines and other oral vaccines [22, 23].

The kinetics of vibriocidal antibody titres in our study showed a significant rise against both *V*. *cholerae* O1 Inaba and Ogawa immediately after vaccination and declined nearly to baseline levels at month 6. A marked rise in antibody titres were then observed at months 36, and 48 post-vaccination. These results indicate that vibriocidal antibodies elicited by the vaccine begin to decline by 6 months post-vaccination. Our data is similar to that of Kanungo, et al [13] in Bangladesh who reported that at 12 months post vaccination the GMTs were slightly higher but not significantly different from the baseline titres. However, the observed increase in antibodies at months 30, 36 and 48 in our study might likely be an exposure to wild type infection, which may have not caused clinical disease possibly due to vaccination, but definitely caused a vibriocidal immune response in this population. The rapid waning of vibriocidal antibodies may be attributable to them mainly consisting of Immunoglobulin M (IgM) [24], which is a marker of recent infection/vaccination and therefore, may not be a true reflection of long-term protection. Though vibriocidal antibodies have been shown to wane quickly after dosing, clinical protection has been shown to last as long as five years [25] in endemic settings and therefore vibriocidal antibodies have been termed as imperfect correlates of protection [26]. In our study, the period between months 6 and 36 was likely to be a critical time for natural transmission and therefore could be the best time for re-vaccination seeing that this area is a cholera hotspot [27]. Therefore, administering a booster dose before month 36 would boost the immunity and protect against infection in endemic areas.

The major strengths of the study is that this is the first immunogenicity study of OCV with a follow up of 48 months; and provides information on pattern of vibriocidal antibody titres in a cholera hotspot in Zambia. The study adds information to the immunogenicity of OCV in Sub-Saharan African specifically the Central part of Africa. This will be useful in providing crucial information on the continued use of OCVs in high-risk areas like this one. However, the study had some limitations. The inability to control for environmental factors that might influence vaccine uptake is a major limitation in this study. Consequently, the high numbers of loss to follow up during the different time points are major limitations. We could not really evaluate seroconversion to full vaccination due to logistical problems in going to collect blood within one-month post vaccination. Earlier studies of live oral cholera vaccines have shown highest titres at 10^th^ day post-vaccination compared to 7^th^ and 14^th^ day [28]. Seeing that vibriocidal antibody wane quickly, we would have obtained sera earlier than day 28 post first and 2^nd^ dose and would have most likely observed a higher magnitude and fold increase in antibody titres. Another limitation of the study is that we did not measure vaccine-specific antibodies and no clinical information was collected in any of the study visit time points to ascertain natural infection. Therefore, the antibody titres observed would have been picked from natural infection by wild type strain.

## Conclusion

OCV Shanchol^™^ was found to be immunogenic in this population. Vibriocidal antibodies elicited by the vaccine after vaccination quickly wane off indicating that vibriocidal antibodies are not a perfect marker of long-term immunity. Protection against disease between months 6 and 36 cannot be explained by using antibody titres only; indicating that there may be other components of the immune system contributing to protection.

## Supporting information

S1 Fig Vibriocidal antibody titres in participants with complete visits (n = 27). Kinetics of vibriocidal log titres expressed as 95% confidence intervals.
(TIF)
