# Supplementary figures and images for: Immunogenicity and waning immunity from the oral cholera vaccine (Shanchol™) in adults residing in Lukanga Swamps of Zambia
Source: PLoS One. 2022 Jan 5;17(1):e0262239. doi: 10.1371/journal.pone.0262239 (PMC8730422; doi:10.1371/journal.pone.0262239)

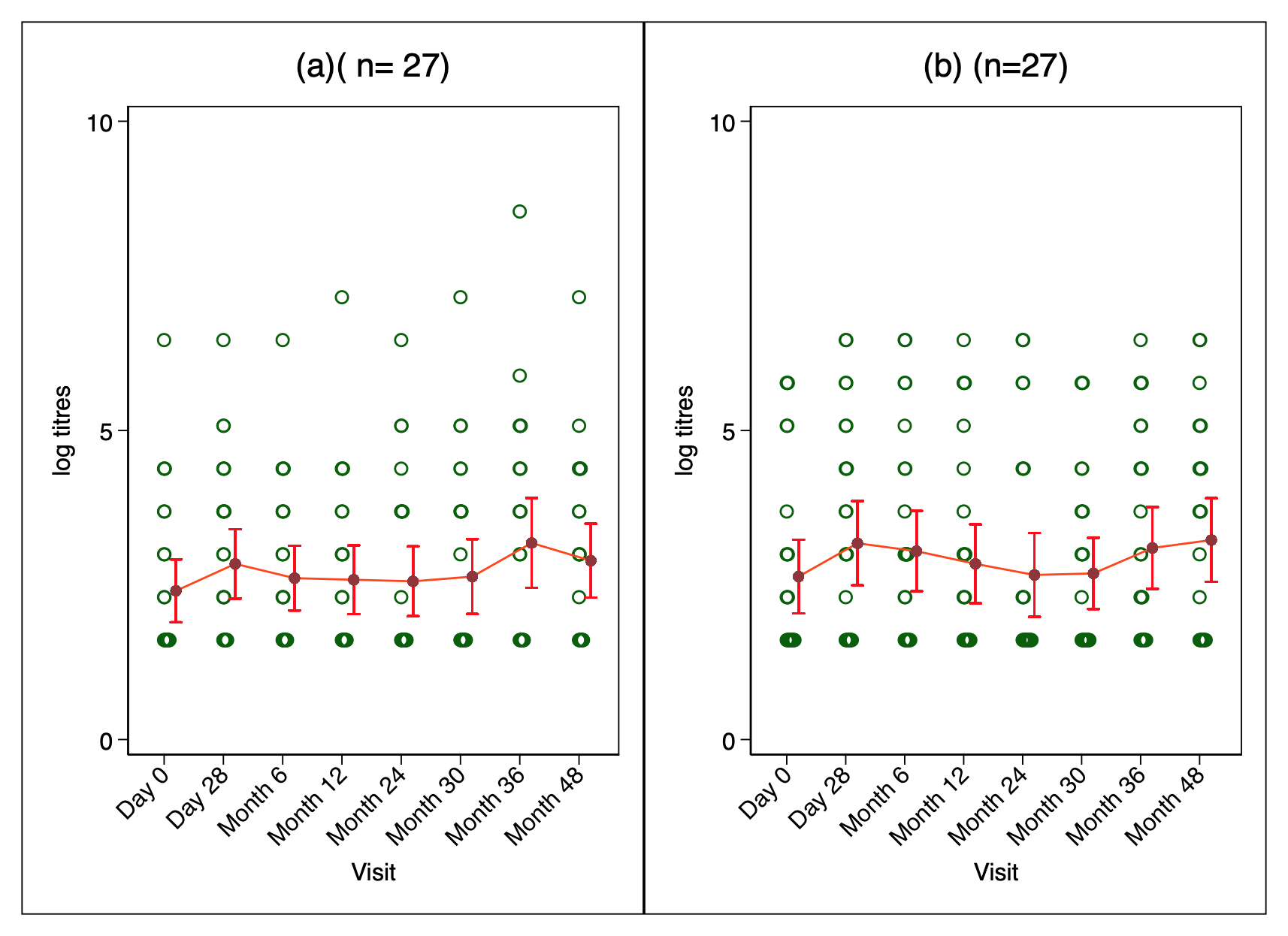

Supplement: S1 Fig — Kinetics of vibriocidal log titres expressed as 95% confidence intervals. (TIF) [file pone.0262239.s001.tif]
